# Microorganism, Carriers, and Immobilization Methods of the Microbial Self-Healing Cement-Based Composites: A Review

**DOI:** 10.3390/ma14175116

**Published:** 2021-09-06

**Authors:** Li’an Shen, Wenlu Yu, Lin Li, Tong Zhang, Ismail Yusuf Abshir, Pingping Luo, Zhuangzhuang Liu

**Affiliations:** 1 Chang’an Dublin International College of Transportation, Chang’an University, Xi’an 710021, China; 2018901447@chd.edu.cn (L.S.); 2018902706@chd.edu.cn (W.Y.); 2018904106@chd.edu.cn (T.Z.); 2 School of Highway, Chang’an University, Xi’an 710064, China; 2020021060@chd.edu.cn (L.L.); yusufabshir22@yahoo.com (I.Y.A.); 3 Key Laboratory of Special Area Highway Engineering, MoE, Chang’an University, Xi’an 710064, China; 4 School of Water and Environment, Chang’an University, Xi’an 710054, China; lpprobert@126.com

**Keywords:** cement-based composites, self-healing, microbial-induced carbonate precipitation, microorganism carrier, microbial immobilization, performance evaluation

## Abstract

Low tensile strength, poor elastic modulus, and complex concrete cracking work condition are almost unavoidable due to the intrinsic brittleness. To deal with concrete maintenance and durability, microbial self-healing concretes have been rapidly developed and widely applied recently. The microbial self-healing can specifically patch fractures as well as boost the concrete structure’s capacity, durability, and permeability. This paper presents the state-of-the-art in the microbe induced self-healing in cement-based composites. The microorganism and carriers were classified according to the working theory and repair effects. Additionally, the precise efficiency and effect of various technologies are also evaluated for microbial immobilization. Based on the literature review and summary from the perspective of microorganism, carriers, and immobilization methods, challenges and further works are discussed.

## 1. Introduction

Cement-based composites, including pastes, mortars, and concretes, are the most used construction materials in the world. However, it is also faced with surface cracks and deep internal micro cracks produced by gradient of temperature, plastic shrinkage, and settlement shrinkage. The cracks will absolutely impact a structure’s engineering performance and shorten service life, resulting in economic losses or even safety incidents [1]. Therefore, it is particularly necessary to patch the concrete cracks in time to keep the cracks from developing and spreading. However, the conventional methods of reconstruction, e.g., surface sealing [2], grouting [3], concrete replacement [4], and the structural reinforcement [5], make it difficult to efficiently repair the interior cracks, as well posing environmental challenges [6].

**Figure 1:**
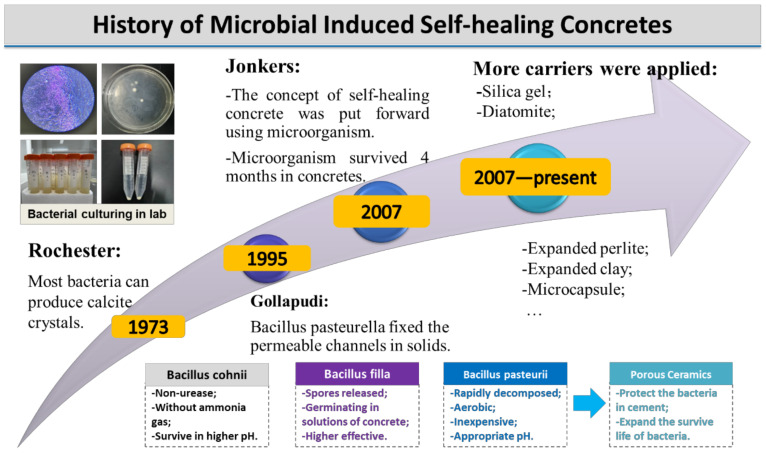
The development of microbial self-healing concrete.

In recent years, an innovative self-healing concrete based on microbial-induced carbonate precipitation (MICP) has become popular since calcium carbonate sedimentation was observed from Bacillus Pasteurella in the 1990s [7]. A lot of progress in the experimental and application side has been made, as shown in Figure 1. Gollapudi reported that the permeable channels could be plugged (resulting in a nil flow rate) based on the process of microbiologically induced mineralization of calcite precipitation [7]. Since then, producing self-healing concrete using microorganisms has been attracting extensive consideration. In 2001, Bacillus pasteurii was fixed in polyurethane foam and added into the concrete, and it was found that the compressive strength of the concrete increased [8]. Ghosh et al. [9] realized that the mineral precipitation and cement mortar strength increased using a thermophilic anaerobic microorganism in which the compressive strength (28d) of cement mortar was improved by 25%. After the concept of microbial self-healing concrete was promoted by Jonkers [10], he implanted bacterial spores in cement stone specimens directly and used the most-probable-number (MPN) technique to estimate the number of viable bacterial cells, which shows it can survive for a period up to 4 months [11]. To deal with the short surviving time of microorganism in concretes, various ways were suggested to protect the bacteria from the extreme environments in concrete. For example, bacteria fixed with silica gel achieved better activity [12], while diatomite was also proved to protect the microorganism in concrete [13].

**Figure 2:**
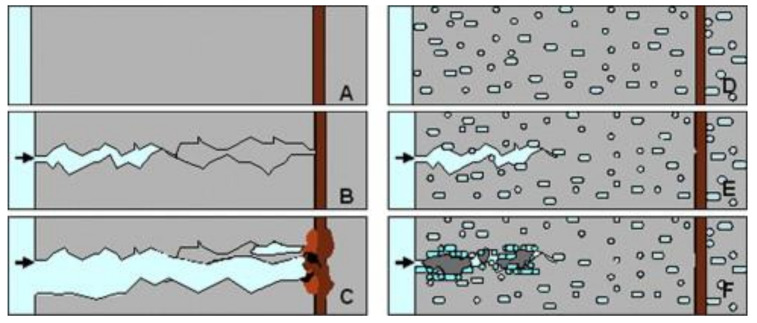
Microbial self-healing concrete model (**A**–**C**): Schematic diagram of conventional concrete cracking; (**D**–**F**): Schematic diagram of self-healing concrete cracking (reproduced with permission from ref. [14]).

The basic illustration and working principle of microbial self-healing concrete is presented in Figure 2. Owing to the brittleness, low tensile strength, alkali-aggregate reactions, and large shrinkage deformation of concrete materials, it unavoidably suffers internal or surface cracks during the preparation and service; exposed concrete structures typically have less longevity, an accelerated corrosion of reinforcement, and degradation of steel reinforcement bars (Figure 2A–C). If the bacterial spores and the nutrients are incorporated in advance (Figure 2D,E), cracks can be sealed by the process of calcium carbonate precipitation after the water infiltrating through cracks (Figure 2F). Therefore, microbial self-healing technology is regarded as an eco-friendly, sustainable, and economical material that has the advantages of intellectualization.

The sustainable development of infrastructure requires the birth and development of concrete composite materials with self-healing capabilities. The significance of self-healing technology is to provide concrete infrastructure with the ability to adapt and respond to the environment, showing great potential and contributing to the creation of various sustainable materials and infrastructure. Due to an enormous amount of capital investment, continuous examination and safeguarding may be tricky to implement, particularly in the case of major construction projects; meanwhile, the development of microbial self-healing technology has become appealing due to the need for minimal labor and low capital expenditure.

To understand the state-of-the-art of microbe-induced self-healing in cement-based composites, the development of microbial self-healing technology is reviewed in this article. The merits and defects of various forms of microbial and carrier materials are addressed, as well as the effects on the concrete remediation effect of the immobilization process. The aim of this paper is to promote the microbial self-healing technology as an alternative solution for ensuring the durability of concrete structures, as well as improve our understanding of the microbe induced self-healing in cement-based composites.

## 2. Microorganism Used in Concretes

Microbial self-healing concrete is based on the bacterial-induced calcium carbonate precipitation [7]. In nature, a lot of bacteria are capable of precipitating calcite (CaCO_3_). According to the way calcium carbonate is produced, the general used bacteria could be primarily categorized into two sorts, i.e., urease bacteria and non-urease bacteria. Most of the urease bacteria and non-urease bacteria can resist the extreme environment inside the concrete and are thus suitable for carbonate precipitation in cement concreting.

### 2.1. Urease Bacteria

Various urease bacteria exist in nature, among which Bacillus pasteurii, Bacillus aerius, Bacillus sphaericus, Sporosarcina aquimarina, Bacillus megaterium, etc. are frequently proposed for the self-healing concrete. Bacillus pasteurii, a Gram-positive bacterium isolated from soil, can grow normally at temperatures ranging from 15 to 37 °C. The urease activities of Bacillus pasteurii is outstanding, which could rapidly decompose urea in the environment into ammonium and carbonate. Bacillus megaterium belongs to Gram-positive bacterium [15]. According to the previous research, it is commonly utilized in the deep sea because of its capability to generate extremely resistant spores in harsh environments. Its survival and growth temperature interval extends largely between 3 and 45 °C [16]. B. sphaericus, Gram-positive aerobic bacterium, forms ellipsoidal spores and is able to produce urease to hydrolyzed urea [17].

**Figure 3:**
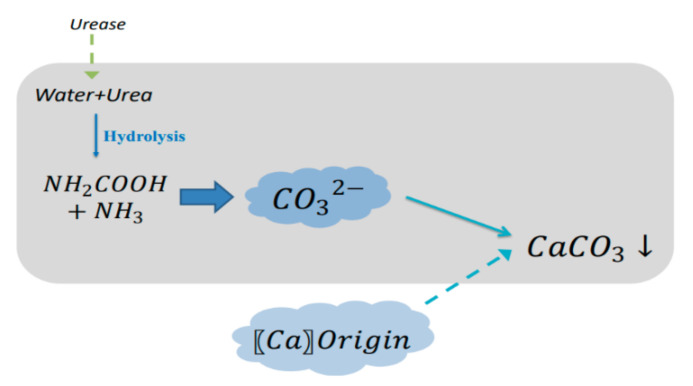
Mechanism of urease-induced precipitation.

Bacterial urease can hydrolyze urea, which will cause CaCO_3_ precipitation and provide self-healing capacity in concretes. In metabolism, urease-catalyzing urea hydrolysis is secreted by urease organisms. As shown in Figure 3, carbamate and ammonia gas are generated in the hydrolyzing of urea molecules with the urease catalysis and then hydrolyzed by carbamate to form carbonic acid molecules and ammonia molecules on their own. Therefore, carbonic acid molecules and ammonia molecules are the end components of urea hydrolyzing. With the assistance of the physiological pH activity of microorganisms, the carbonic acid protons are dissociated, and the water molecule protonates the ammonia molecule, which contribute to a rise in pH value. Calcium ions can interact with carbonic acid and precipitate to form crystallization under the atmosphere of high concentration of carbonic acid and solid alkali (Figure 3). Table 1 summarizes the calcium carbonate precipitation reaction formula caused by the urease bacteria [18,19].

**Figure 4:**
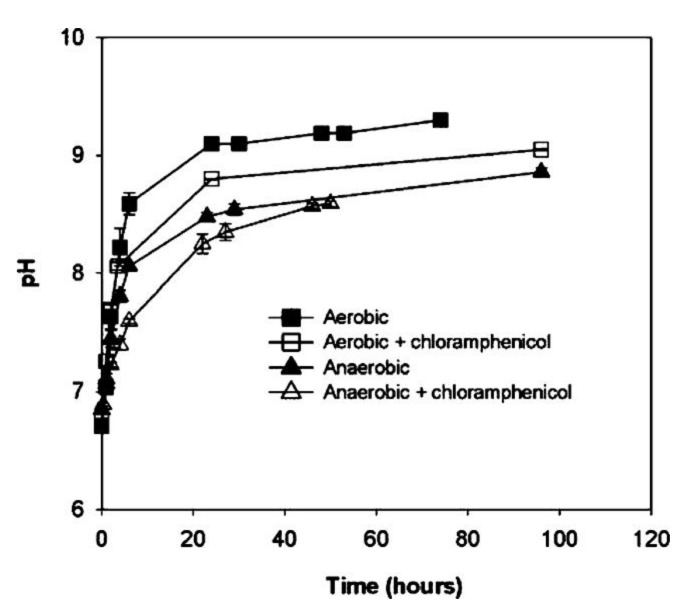
Changes in pH for *S. pasteurii* grown aerobically and anaerobically with and without the addition of chloramphenicol (reproduced with permission from ref. [25]).

According to the research to induce the precipitation of calcium carbonate based on Bacillus cohnii, Bacillus pasteurii, and Bacillus sphaericus, in terms of the repaired width of cracks from microorganisms, the Bacillus cohnii self-healing performance in concrete specimens is more effective. The crack that Bacillus cohnii repaired had a width of 0.56 mm [20]; meanwhile, the compressive strength was improved by 30% [21]. The Bacillus pasteurii gained the highest urease activity and the maximum restored crack width of 3.8 mm under the pH value of 6.8–8.0 [22]. The maximum diameter of cracks that can be repaired by Bacillus sphaericus is 0.97 mm [23]. In terms of the appropriate pH for the microorganism, the Bacillus pasteurii is aerobic, inexpensive, and not able to live in the rigorously regulated anaerobic environment. However, there are numerous research studies using Bacillus pasteurii [24,25,26] because it can maintain activity under a pH value of 9.25 in positive temperature and aerobic conditions (see Figure 4). Moreover, the best pH for Bacillus sphaericus-induced carbonate precipitation is 7.0, which is part of a genus of bacteria that does not withstand alkalinity and has higher temperature adaptability [27,28]. Additionally, the time required and process to ferment bacillus pasteurella is not too complicated. Simultaneously, spherical calcium carbonate crystals are effortlessly formed under the high concentration of bacterial liquid, whereas the ratio of cuboid to aggregate crystal gradually increases with the decrease of concentration [29]. However, the urease behavior of Bacillus pasteurii is slightly decreased at lower temperature [30]. The calcium carbonate content rose over time when Bacillus sphaericus was used to produce the precipitation of calcium carbonate, but no clear upper limit was given [31]. Table 2 summarizes the optimum pH value and maximum repaired crack width of different microorganisms.

### 2.2. Non-Urease Bacteria

As for the non-urease bacteria, Bacillus pseudofirmus, Bacillus cohnii, Bacillus halodurans, Bacillus mucilaginous L3, Enterococcus faecalis, Geobacillus stearothermophilus, Bacillus subtilis, etc., are widely studied as non-urease bacteria inducing calcium carbonate precipitation. Bacillus subtilis is a Gram-positive bacterium that forms oval or cylindrical spores [36]. Numerous bacillus subtilis are used in agriculture and in some medicines, therefore it is not detrimental to human health. The Bacillus pseudofirmus hydrolyze urea into NH_3_ and CO_2_ by using urease produced by themselves. In alkaline environment, the pH value of the solution with the increase of NH_3_ and CO_2_ exists in the form of CO_3_^2−^.

For non-urease bacteria, they will transform organic acids to form calcium carbonate precipitates through their own vital activities under oxygen-containing conditions. Calcium lactate or calcium acetate are often added to nutrients that non-urease bacteria can eventually convert to calcium carbonate. Equation (8) shows the reaction in which calcium lactate works as a carbon source [11].

CaC_6_H_10_O_6_ + 6O_2_ → CaCO_3_ + 5CO_2_ + 5H_2_O (8)

**Figure 5:**
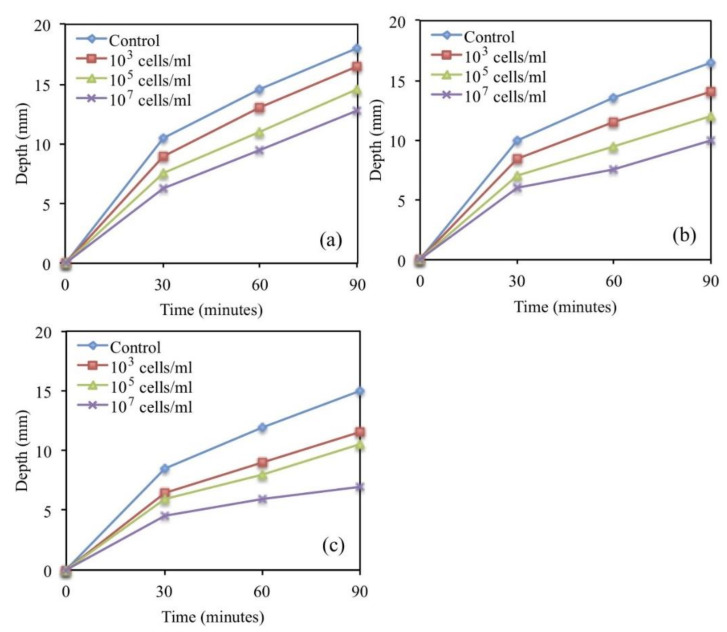
Increase in water penetration depth w.r.t. time for control and microbial mortar samples (**a**) 3 days, (**b**) 7 days, and (**c**) 28 days (reproduced with permission from ref. [37]).

**Figure 6:**
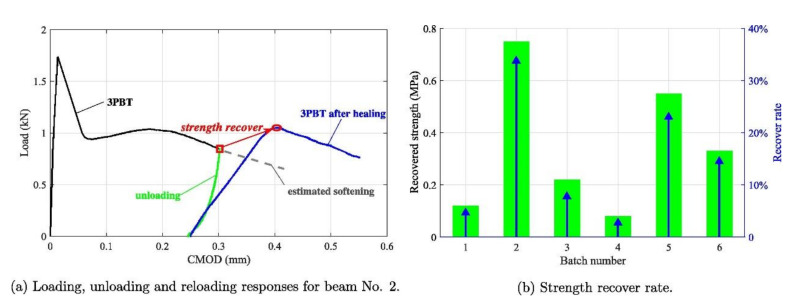
Strength recovery for self-healing concrete (reproduced with permission from ref. [38]).

Most microorganisms are intolerant to alkaline environments. The Bacillus pseudoadamentosa has an exceptional ability to adapt to the alkaline conditions where the surviving pH value can be up to 11.0 [30]. At 10 pH condition, the growth of Bacillus pseudofirmus is the fast, indicating the most alkali-resistent behavior. Based on its ability to survive in a more alkaline environment, numerous scholars studied it and reported that the maximum healed crack diameter by Bacillus pseudofirmus was 0.46 mm [33]. Sharma et al. [32] suggested that the spores of Bacillus pseudofirmus are simple to shape and the healed crack diameter is 100 times the width of the calcite crystals. Tiwari et al. [30] observed that the calcium carbonate content produced by Bacillus pseudofirmus had reached 94.32%, and its mineralization potential was higher. For Bacillus subtilis, it has a higher yield of biofilm on the bacterial surface and better repair efficiency with a median crack healing diameter of 1.8 mm. In addition, Bacillus subtilis results in a 22% increase in compressive strength and a 7% increase in flexural strength [34]. In addition, Bacillus subtilis has a good performance in the mechanical properties of the repaired concrete. It decreases the rate of water penetration, and the maximum crack width it could repair reached 0.3 mm (as Figure 5). It was observed that the microbial repair in the crack area mainly occurred a few days after the crack formed [37]. After the concrete specimen had undergone the first three-point bending test (3PBT) to produce cracks and curing for 28 days, another 3PBT was carried out to determine the strength recovery rate due to microbial self-healing. For the healing process, the cracks are filled with visible white mineral calcium carbonate. After 28 days of healing, the average recovery strength of the concrete specimen was 14% (as Figure 6) [38].

Overall, for the case of urease bacteria, it makes use of a series of reactions by itself to precipitate the CaCO_3_ crystals, whereas non-urease bacteria makes use of reactions by transforming organic acid into CaCO_3_ precipitation. However, the non-urease bacteria would not produce harmful gases such as NH_3_ and contributes an eco-friendly environment role in concrete.

Microorganisms play an effective role in fixing concrete and preserving the mechanical properties and durability of concrete during the process of healing micro-crack concrete by microbial self-healing technology. Although a wide range of microorganisms can heal cracks greater than 300 μm under the right conditions, different types of microorganisms may have distinct healing effects. In microbial self-healing concrete, the healing results are influenced by significant factors such as whether to have carriers, how to use carriers, and how bacterial interacts with carriers.

## 3. Carriers to Protect Microorganism

### 3.1. Porous Inorganic Materials

Inorganic porous carrier materials typically have porous surface properties and good absorption of water. The rich microporous structure will provide adequate room and sustain excellent connectivity for the growth and metabolism of microorganisms. Meanwhile, the comparatively thick surface can reduce the penetration of high alkali substances, which can significantly increase the tolerance of concrete bacteria.

The lightweight porous aggregate carrier often belongs to the inorganic porous material; extended perlite, porous ceramist, diatomite, zeolite, pelelith, etc. are typical porous light aggregate carriers. These natural inorganic porous products are commonly used as a promising material in self-healing concrete for sustainable infrastructure, because they have strong compatibility with concrete and the characteristics of higher bacteria content, lower incorporated amount, and cost-effectiveness.

**Figure 7:**
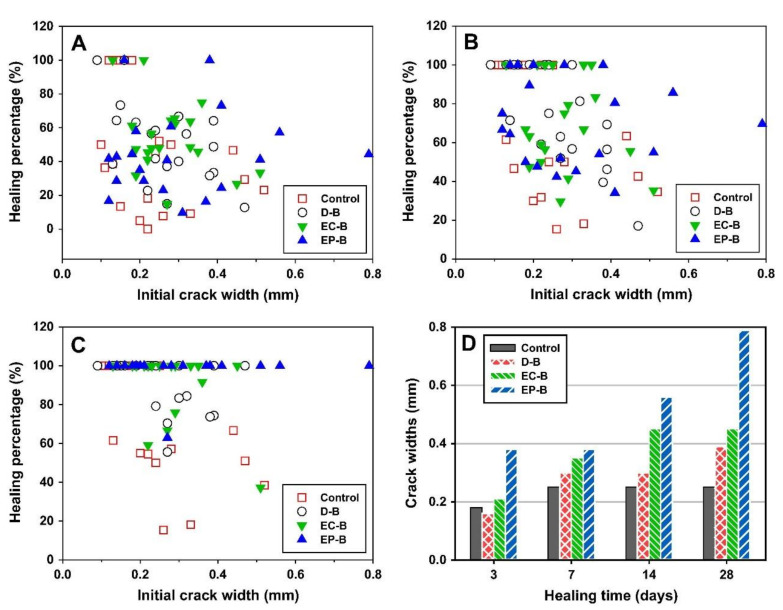
The relationship between the percentage of crack healing and the initial crack width when the concrete specimen is healed at 3 days (**A**), 7 days (**B**), and 28 days (**C**). The maximum value (**D**) of the healing crack width at different stages of healing time (reproduced with permission from ref. [39]). Note that the Control is without bacterial spores; D-B was directly introduced bacteria without any protective carrier; EC-B was used with EC particles as protective carriers; EP-B used EC particles as protective carriers.

The immobilization of bacteria inside expanded perlite (EP) shields bacteria from concrete’s high-pH climate and increases the self-healing to a large extent, enhancing the bending properties of concrete. Meanwhile, most of the bacterial treatments are restricted to the surface only up to few micrometers, and the use of expanded perlite can increase the healing rate in the deeper position of cracks. Numerous 100 mm cavities were found in expanded perlite particles using the Field Emission Scanning Electron Microscope (FESEM) and X-Ray Diffraction (XRD), which could provide free oxygen sufficient for bacteria inside the concrete and attachment space for the fixed bacteria to carry out their metabolic activities. At the same time, it can also make bacteria exposed to small amounts of water when cracks appear; thus, the expanded perlite exhibits excellent characteristics associated with high porosity and highwater absorption. The specimens incorporated with EP particles for B. Cohnii exhibit completely healed crack widths of up to 0.79 mm, which is larger than the value of 0.45 mm for specimens incorporated with expanded clay particles (as Figure 7) [39]. A recent advancement in the bacterial immobilization inside of expanded perlite is the use of sugar-coated expanded perlite as a bacterial carrier for crack-healing concrete application, the EP particles immobilized with bacterial spores were wrapped with a low-alkaline material, and the healed width can reached 1.24 mm [40].

Diatomaceous earth (DE) particles are found to have a protective effect in a high-pH cement environment with porous properties; they have a strong capacity to sorb bacterial cells on the surfaces and provide a new microenvironment, which enables bacteria keep degrading urea [13]. The findings of the experiment revealed that DE immobilized bacteria can still sustain such enzyme activity and totally fix cracks with a width of 0.15 to 0.17 mm even in a high pH of 12.5 cement area. The amount of DE power is very significant for latter application because DE particles have a strong capacity to absorb water, and after adding DE particles into the mortal paste, the mortal paste would be very dry and influence the workability.

To improve the loading content of the protective carriers, different pretreatment procedures, such as alkali erosion and sintering treatments, on lightweight porous aggregate were carried out beforehand. Ceramic particles as a carrier of bacteria were treated by NaOH solution with concentrations of 0.5 mol/L, 1.0 mol/L, and 1.5 mol/L to increase the number of loading bacteria [41]. It is observed that the porous ceramic content has an influence on the concrete compressive strength; with the increasing in the porous ceramic content, the concrete compressive strength first marginally increases and then decreases, and 55% of the station’s aggregate volume is the optimum porous ceramic material. Meanwhile, it can be found that the cracks of the concrete sample are filled with calcium carbonate deposition products after 28 days curing for self-healing, and the self-repair effect is apparent. The compressive strength recovery rate is about 63%, and the repairable maximum width is about 0.51 mm. Zeolite is widely utilized as an immobilization material in wastewater treatment on the basis of its widespread occurrence in nature, and it can also be used to immobilize microorganisms that can reduce the cost of self-healing concrete to a certain extent.

**Figure 8:**
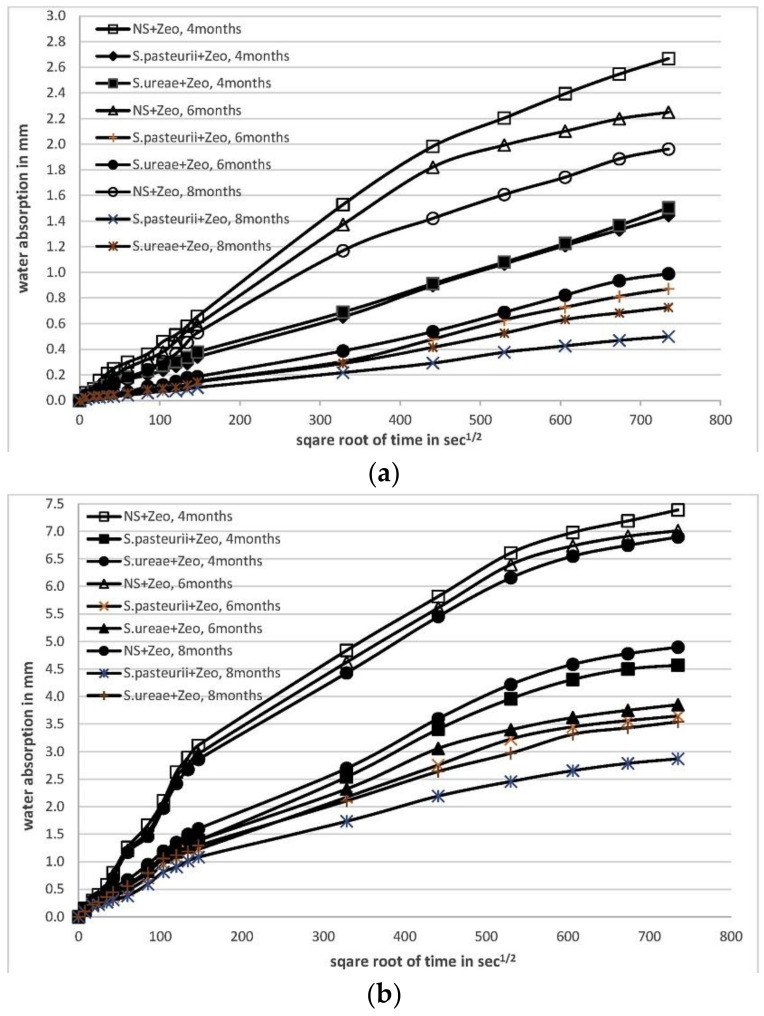
(**a**). Water absorption (mm) versus square root of time (sec) in cracked specimens. (**b**). Water absorption (mm) versus square root of time (sec) in normal mortar with holes (reproduced with permission from ref. [42]) (NS: Nutrient solution; *S. pasteurii:* Sporosarcina pasteurii; *S. ureae*: Sporosarcina ureae; Zeo: zeolite).

Bacillus pasteurella was found to survive in concrete (pH = 12) with the protection of zeolite and to produce calcium carbonate crystals after self-healing evaluation on crack-healed specimens at ages 4, 6, and 8 months cured in water, and the compressive strength improved with the addition of bacteria (10%) [42]. However, as Figure 8 shows, the presence of bacteria significantly reduced the water absorption of the specimens, particularly during the first four months of treatment. However, the rate of water absorption did not much improve in the later age, indicating that the capacity for self-healing was limited.

Pelelith can also function as a bacterial carrier to protect bacterial spores and investigate the repairing of concrete cracks. Compared to other carriers, the surface of pelelith is positively charged and its physical and chemical characterization are stable, making it more compatible with microorganisms and conducive to the settlement of calcium carbonate. Wu et al. [43] investigated the repair of concrete cracks using pelelith as bacterial vehicle, conducted experiments on concrete specimens such as unregulated compressive strength and ultrasonic wave, and compared and evaluated the efficacy of the immobilized pelelith bacterial system and conventional methods. In this repair process, concrete specimens with crack widths of 1.5 mm and 2.0 mm could be fully sealed after 20 days of repair, with resistance recovery rates of 19.68% and 15.51% respectively, and the repair impact near the upper surface of the specimens was higher.

**Figure 9:**
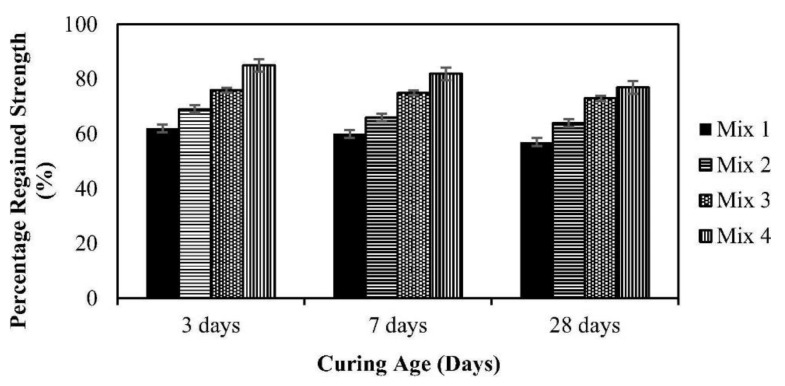
Percent regain of compressive strength w.r.t time at different pre-cracking age (reproduced with permission from ref. [44]) (Mix 1: reference mix without bacterial cells; Mix 2: bacterial cells were incorporated directly through makeup water; Mix 3: bacteria cells were immobilized through RCA; Mix 4: bacterial cells were incorporated through RCA and 50% virgin FA).

Considering the factors such as local availability, cost, and protect impact of bacterial cells, advanced study focuses on bacteria that are immobilized via recycled coarse aggregate (RCA) and virgin fine aggregate (FA). It was found that using CA and 50% virgin FA as bacteria immobilizers exhibited the most effective crack repair method, which can heal a maximum crack width of 1.1 mm and restore 85% of the compressive strength while minimizing energy consumption and effectively lowering anthropogenic emission (as shown in Figure 9) [44].

### 3.2. Organic Materials

Organic material can be split into natural organic material and artificial organic material. Natural organic compounds have greater stability with microorganisms relative to other materials. However, under certain conditions, some natural materials are easy to decompose by microorganisms and have poor strength, which has a certain effect on the strength of concrete after repair. Artificial organic materials provide stable chemical and physical properties, a high capacity for anti-microbial decomposition that can offer long-term protection for microorganisms such as polyurethane porous foam, microcapsules, etc., in a strong alkaline setting.

**Figure 10:**
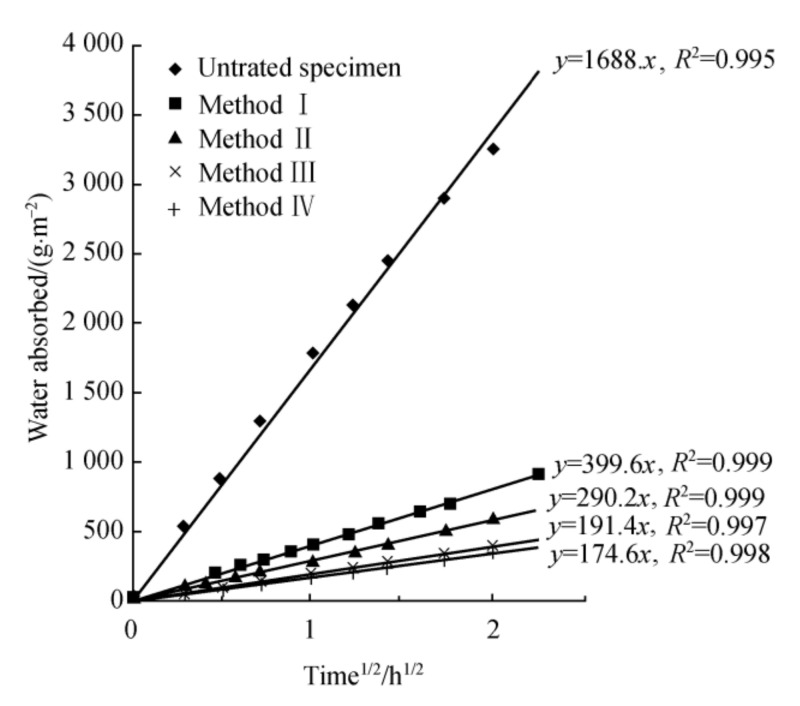
Influence of different lamination conditions on capillary water absorption capacity of cement paste specimens (reproduced with permission from ref. [46]). (Method Ⅰ: Spraying method; Method Ⅱ: Immersing method; Method Ⅲ: Brushing with sodium alginate; Method Ⅳ: Brushing with agar).

To sustain a high degree of bacterial enzyme activity and provide a microenvironment for bacterial growth, the digestion of urea, and the deposition of CaCO_3_, natural organic support materials, agar, and odium alginate were used to immobilize the bacteria in Qian’s experiment [45,46]. The result revealed that the overall distance that can be repaired is 0.1 mm, however, the addition of the carrier greatly reduces the water absorption capacity of the concrete sample by 75% to 90%, and after completing the whole repair process, the pressure strength has been lowered to some degree (as shown in Figure 10).

**Figure 11:**
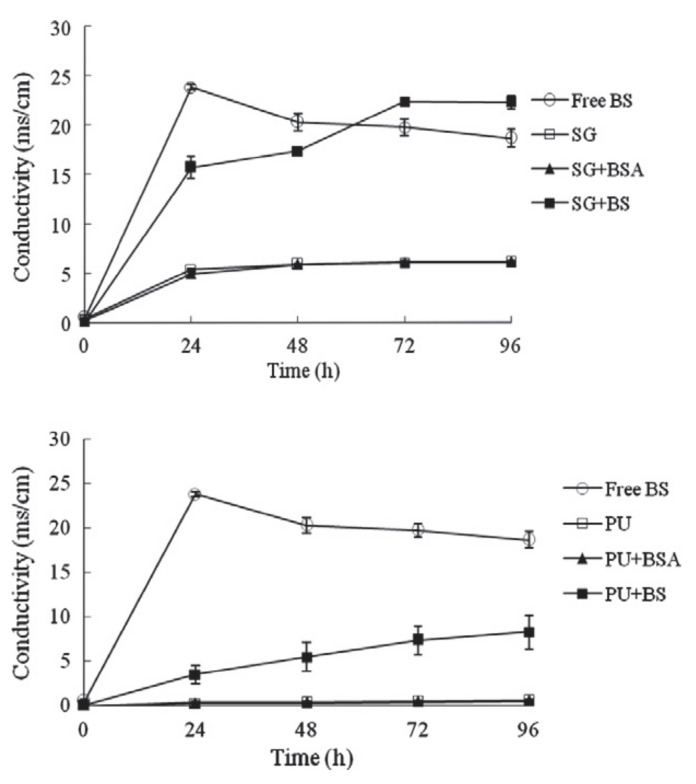
Ureolytic activity of un-immobilized and immobilized (silica gel or polyurethane) bacteria (reproduced with permission from ref. [12]) (Free BS: un-immobilized bacteria; SG: added with silica gel; SG+BSA: added with silica gel immobilized dead bacteria; SG+BS: added with silica gel immobilized living bacteria; PU: added with PU foam; PU+BSA: added with PU immobilized dead bacteria; PU+BS: added with PU immobilized living bacteria).

Polyurethane (PU) is widely used as a waterproof material. The possibility of using silica gel or polyurethane as a carrier for bacterial protection was investigated by Wang [12]. The activity of bacteria can be reflected by the quantity of calcium carbonate precipitated in the carrier, and the results show that bacteria immobilized by silica gel perform better activity (Figure 11). The specimens with polyurethane as the carrier show greater self-healing performance that much exceeds the recovery capacity of fixed microbial specimens with silica gel.

**Figure 12:**
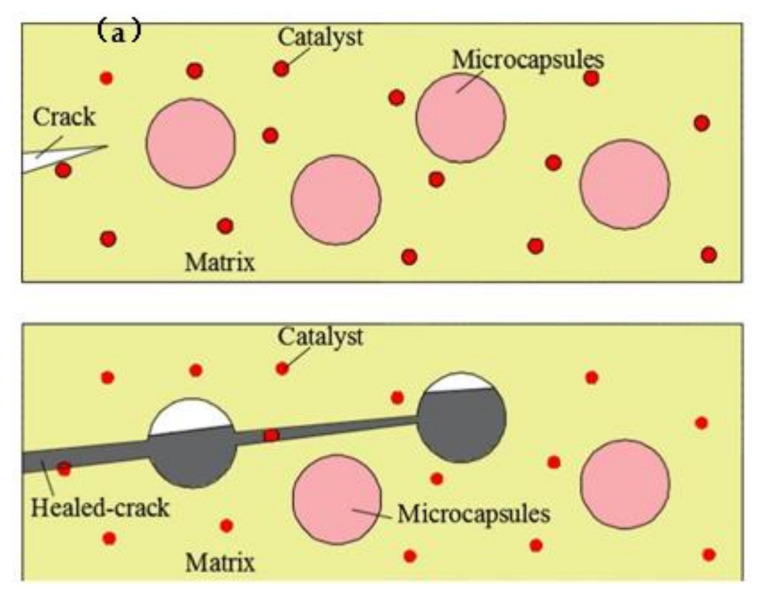
The restoration model of microcapsule technology (reproduced with permission from ref. [49]).

**Figure 13:**
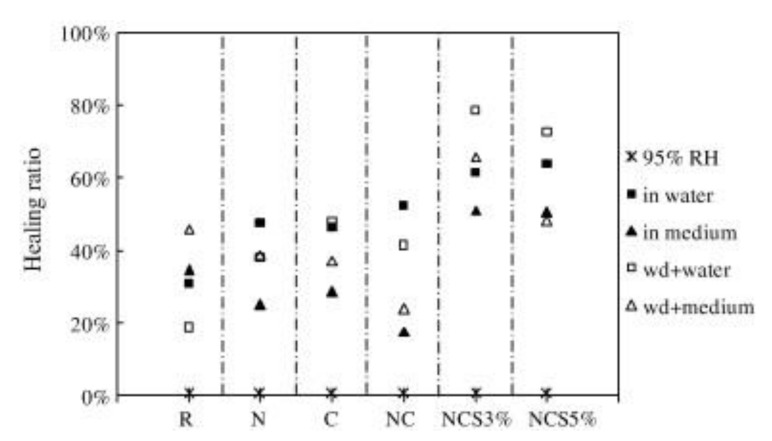
Healing ratio in each specimen under different incubation conditions (reproduced with permission from ref. [48]) (non-bacteria series: R, N, C, NC; bacterial series: NCS3% and NCS5%; wd: wet–dry cycle).

Microcapsule is an artificial organic material, and this self-healing system has been developed mainly in polymers and composites, with the potential to provide bacteria with all around protection. Some microcapsule spores are used by researchers to isolate the outside world, which is helpful for resting and lurking spores [47]. Spores began to transform from dormant to activate state when cracks appeared, capsules were broken and reached the nutrients and began self-healing in the right environment (Figure 12). Wang et al. [48] observed that the healing rate of microcapsule specimens with microorganisms (48–80%) was higher than that of bacteria-free specimens (18–50%); the median crack healing width of the microcapsule specimens was 0.97 mm, which was about four times (the maximum 0.25 mm) of the specimens without bacteria (Figure 13). The overall permeability was around 10 times lower for microcapsule specimens than for non-bacterial series. The addition of microcapsules reduces the spatial structure of voids, thereby reducing water absorption, even while it had no obvious influence on the degree of motor cement hydration. For economic and safety purposes, it is also recommended that the addition of biological microcapsules should be restricted to less than 3%.

**Figure 14:**
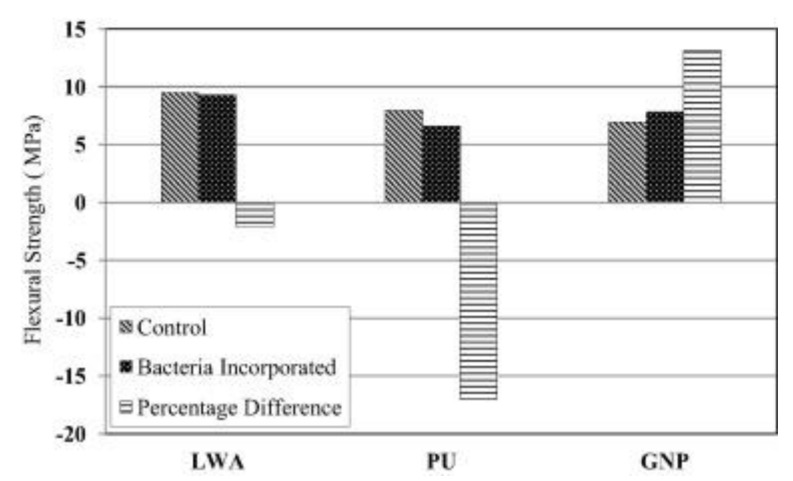
Comparison of flexural strength in lightweight aggregates (LWA), polyurethane (PU), and graphite nano-platelets (GNP) incorporated concrete (reproduced with permission from ref. [50]).

Nanometer materials also contained artificial organic material which, due to the nanoscale size of the carrier, has certain advantages in improving the healing efficiency and performance of concrete. Khaliq et al. [50] have shown that graphite nano-platelets have better concrete self-repair potential. The size of this sheet of graphite nanometer allows the uniform delivery of the curing agent, which will better facilitate concrete’s self-healing mechanism. As Figure 14 shows, unlike the lightweight aggregate carrier, graphene nano-sheets can increase the flexural strength of concrete; therefore graphene nano-sheets are helpful for reducing concrete cracks. Some limitations also need to be taken into consideration, which are necessary for the application of this technology at commercial scale, because nanometer material is relatively expensive.

As summarized in Table 3, carriers affect the strength and crack width of cement or concretes. The selection of the carrier should be based on the specific conditions and intended purposes. There are several environmental factors that need to be considered while developing the self-healing system, such as temperature, moisture access, and external ions. Inorganic healing agents are preferred for concrete structures in water-rich regions or humid environments, whereas organic healing agents are preferred when the concrete structures have no direct access to water. Organic healing agents exhibit better healing performance of regaining the mechanical strength of original concrete, whereas inorganic healing agents show greater potential of regaining the tightness of original concrete [51,52]. It is noteworthy that the inorganic healing products have great potential in regaining tightness to resist the ingress of deicers, thus inorganic healing agents are preferred in cold and marine regions for concrete infrastructure [53].

Although using the carrier would extend the life of microorganisms in an extremely alkaline environment, but the successful running time of existing bacteria is still inadequate relative to the real engineering application. Simultaneously, the inclusion of most carriers can affect the compressive strength of concrete samples to some degree, especially when multiple immobilized microorganisms die; thus, the resulting compressive strength of concrete will not fulfill the actual specifications for engineering. While microorganisms may be shielded from changes in the external environment and retain their biological action by the inorganic porous material carrier, the structure also creates considerable transfer resistance to the activities of bacterial existence. In addition, organic carrier materials have strong stability with microorganisms, but they are vulnerable to aging and decrease the repair effect. Therefore, the mechanical properties of carrier materials, their microorganism compatibility, and the resulting degree of concrete need to be further researched.

The carrier mechanical properties can be changed by the qualitative alteration of surface groups or microporous composition of carrier materials, but it may come with a higher cost. Microcapsule technology is a great breakthrough in the future development direction of the carrier, it can effectively resist the mechanical force of concrete mixing, keep the microorganism active, and it has almost the highest healing rate among all the healing agents. The development direction of organic carrier materials in the future can be focused on the preparation of capsules, the mechanical properties of capsules, and the created empty space after healing. Meanwhile, the scale of the carrier can also be a great innovation. The incorporation of micro-scale carriers has a negative effect on the mechanical properties of concrete, while metal nanoparticles have a positive effect on the mechanical properties of concrete and its high compatibility with the concrete matrix. Therefore, the development of a nanoscale bio-concrete carrier is very promising and necessary. To facilitate the production and deployment of self-healing concrete technologies, we need pay more attention to find productive carrier materials and acceptable doses.

## 4. Microbial Immobilization Methods and Procedure

### 4.1. Methods for Microbial Immobilization

A lot of microbial immobilization methods have been reported to immobilize the microorganisms into carriers to make microbes highly dense, to preserve microbes’ biological activity, and to proliferate microbes under ideal conditions [54]. In microbial-induced carbonate precipitation (MICP), the predominantly used immobilization methods include adsorption treatment methods (ATM) [13,20,43,55,56], the microcapsule-embedding method (MEM) [48,57], and the vacuum impregnation method (VIM) [39,58,59]. The ATM is usually utilized to fix the microorganisms on porous carriers. This method achieves an equilibrium position where the liquid mixture is in full contact with the absorbent (i.e., porous carriers) for a fixed time to absorb microorganisms on the pore surface. The MEM refers to the immobilization of microorganisms embedded in microcapsules of polymeric semipermeable membranes. The diameter of the immobilized microencapsulated enzyme is usually between several microns and several hundred microns, which is suitable for the immobilization of small molecular substrates and products of enzymes. The VIM refers to impregnating the microorganisms into the carrier by negative pressure under vacuum conditions, thereby improving the properties of the materials. Under vacuum conditions, the negative atmospheric pressure makes the gas escape, and the bacterial solution will be pressured (infiltration and diffusion) into the pores of carriers through the channel gas escaping.

**Figure 15:**
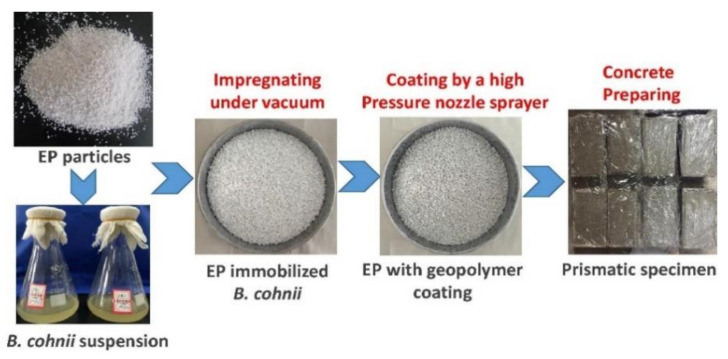
Bacterial strain immobilized by vacuum impregnation method (reproduced with permission from ref. [39]).

**Figure 16:**
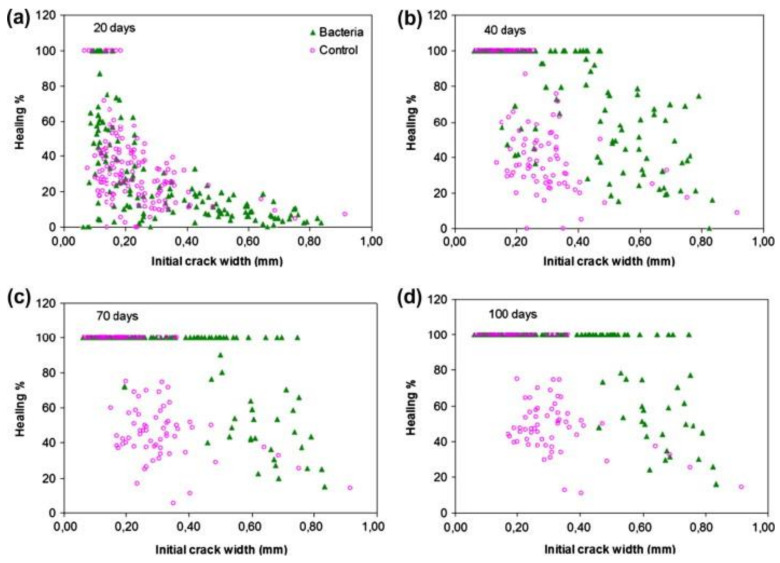
Crack healing percentage as a function of the initial crack width for bio-chemical agent-based and control mortar specimens. For each, results from five cracks (150 measurements) are plotted after immersion time of (**a**) 20 days, (**b**) 40 days, (**c**) 70 days, and (**d**) 100 days (reproduced with permission from ref. [58]).

Currently, the vacuum impregnation method is one of the most commonly used technologies. Figure 15 shows one successful experimental process for VIM made by Zhang et al. However, it is necessary to pay attention to the rigor of the experimental operation process of VIM. The vacuum, dry process, and the proportion of substance in impregnation will affect the property of MICP. However, in vacuum impregnation, the order of impregnation selected by different scholars is not always the same. Some scholars prefer the order of impregnation as the calcium source, nutrient, and microorganism, while others prefer the order of microorganism, calcium source, and nutrient. Nevertheless, it is undeniable that each impregnation must be followed by drying, and drying is usually in an oven at a certain temperature for a certain period of time. Considering the survival rate of microorganisms, the drying temperature should not too high and the drying time is usually several days until the weight is constant. Otherwise, different scholars used different kinds of calcium sources, nutrients, microorganisms, and carriers. However, most of the repair results of scholars showed that the materials they chose could repair cracks of a certain width or depth. Zhang et al. [39] proved that the diameter of the completely healed crack was up to 0.79 mm after 28 days of healing. Ren [55] found that the deposition depth could reach 1.0 mm, and the average 30-day area repair rate was over 95%. Wiktor et al. [58] healed only 0.46 mm wide cracks on average (Figure 16).

**Figure 17:**
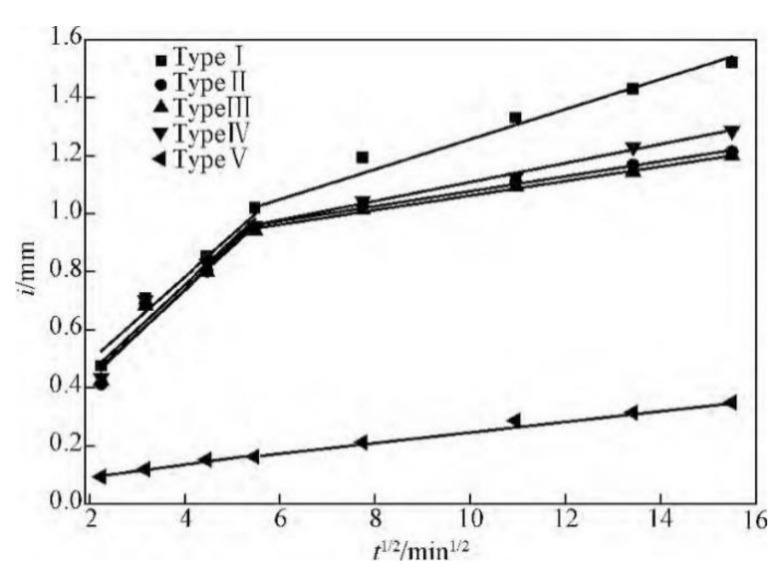
Water absorption of specimen surface with different film-covering method (reproduced with permission from ref. [60]) (Type I: Untreated; Type II: Agar only; Type III: Bacteria and mixture; Type IV: Agar and mixture; Type V: Bacteria immobilized by agar and mixture).

The brushing method also has been used in microbiological self-healing concrete. The brushing method usually refers to the bacterial suspension and calcium source; nutrients are respectively brushed on the surface of the cement specimen, which can achieve the effect of repairing the specimen surface cracks. Among them, Qian and Ren [60] smeared an agar layer on the surface of cement specimen at first. Then, they repeatedly smeared and sprayed bacterial suspension, calcium source, and nutrients. Finally, they found that concrete specimens with crack widths less than 100 μm could be repaired, and the capillary water absorption coefficient was reduced by 86% at most (Figure 17). However, the brushing method only has a certain repair effect on the surface crack because it only applies the necessary substances to the surface of cement stone. It cannot repair the internal micro cracks of concrete, and the survival time of microorganisms on the surface of cement stone is limited. Thus, it can only be used to repair some existing cracks.

**Figure 18:**
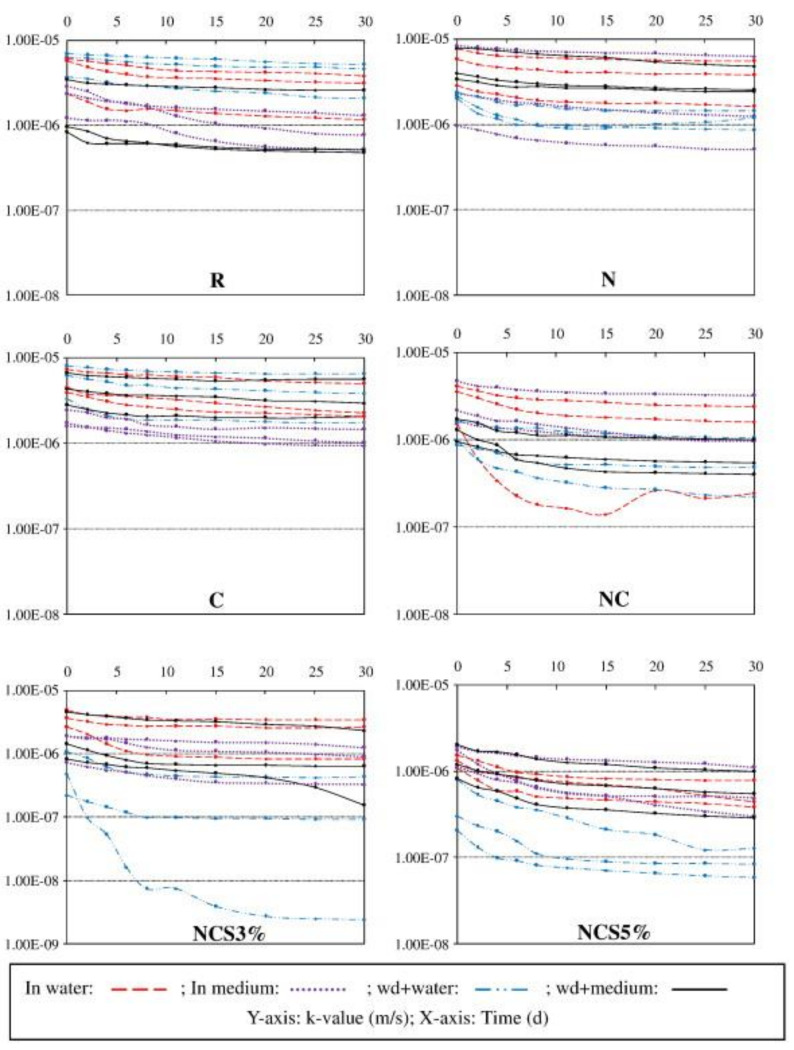
Water permeability of the specimens during the test periods. (reproduced with permission from ref. [48]) (non-bacteria series: R, N, C, NC; bacterial series: NCS3% and NCS5%).

ATM is often used with a shaker to mix the bacterial suspension with the carrier; then, it mixes the mixture with nutrients before adding it to the concrete. The adsorption experiment conducted by Wang et al. [13] showed that the specimen cracks below 0.17 mm had been completely filled, and the precipitation in the crack is mainly composed of a small amount of urea or calcium nitrate crystals containing calcium carbonate. MEM is a popular method in the field of microbial self-healing at present. It embeds all necessary substances in microcapsules that can effectively protect microorganisms and nutrients. Finally, it can effectively reduce the water permeability, improve the strength of concrete, and repair the crack with a width of 970 μm [48] (Figure 18).

### 4.2. Immobilization Procedure

In the process of microbial self-healing concrete, the immobilization procedure is also very important. The success rate and practicality of the microbial and carrier mixture would be influenced by the rational choice and implementation of the immobilization method. The combination of the microorganism and the carrier, which performs the protective role of the carrier, increases the survival rate of the microorganism, and eventually reaches the complete fix, will be supported by the proper method of immobilization. The selection of the immobilization approach only has some effect on the experimental results relative to the microbial species and carrier species that play a decisive role in the maximum repair width of concrete. Thus, when using same immobilization method, there may be a noticeable change in the maximum repair width of the concrete. Otherwise, if using the same type and quantity of microorganism, carrier, and nutrients, due to the different immobilization methods, in which the immobilization process, the immobilization effect, and the mechanism are different, there may be a various maximum repair width (seeing Table 4), compressive strength, flexural strength, water permeability, and chloride ion permeability. However, on the premise that the immobilization method is reasonable and the immobilization process is precise, these influences will be negligible relative to the changes of microorganism and carrier species.

## 5. Evaluation and Practice of the Microbial Self-Healing Cement-Based Composites

### 5.1. The Impact Factors of Self-Healing Capacity

In addition to the detailed description of microbial species, carrier types, and microbial immobilization methods that would impact the self-healing capacity, the working conditions especially temperature, water, the addition of nutrients, and even pressure are also important. The spores need to revive when the crack is producing. However, at different temperatures and different amounts of water, the spore recovery rate would be different, which will affect the calcium carbonate production rate. Furthermore, the amount and types of nutrients also should be considered; the addition of nutrients could promote the growth and mineralization efficiency of bacteria, as well as their ability to produce healing product. As result self-healing effect of concrete cracks could be improved. Moreover, in laboratory research, pre-cracking methods are also an important influencing factor. Many scholars use an electro-hydraulic servo testing machine, compressive servo testing machine, and automated bending compression testing machine to construct cracks. Each pre-cracking method will produce different crack widths and depths. When the crack is too large, the repair ability of microorganisms may not be enough to allow the crack to accumulate calcium carbonate, which makes the experiment unable to accurately evaluate the self-repair efficiency. Low pressure in a high-altitude area will affect the performance of cement mortar [63]. It is necessary to verify the effect of microbial self-healing under this condition separately.

### 5.2. The Evaluation Index of Self-Healing Capacity

There are two main categories to evaluate the self-healing capacity, which include the material detection method and physical inspection method. For the material detection method, scanning electron microscope (SEM), energy-dispersive spectroscopy (EDS), X-ray diffraction (XRD), and thermogravimetric analysis (TGA) are very common ways to investigate the morphology, energy distribution, and composition of crystals precipitated in cracks. SEM is a kind of observation method to obtain the microscopic morphology characterization of material by scanning with a high-energy electron beam. EDS uses X-ray photons to map the size distribution of energy. XRD is a technique for determining the crystal structure based on the diffraction patterns obtained from the emission of electromagnetic waves. TGA is a method of measuring the mass of a substance in relation to temperature or time. In addition, the physical inspection method concentrates on testing the self-healing capacity of concrete specimens, which includes compressive strength, flexural strength, water absorption, water permeability, durability, maximum repair width, maximum repair depth, etc. Meanwhile, the chloride penetration resistance can also be used to evaluate the self-healing capacity. Fahad et al. innovated the steady-state migration test under higher potential, and the self-healing capacity showed it can provide a more precise result [64].

### 5.3. Practical Application

With the development of microbial self-healing technology, more and more microbial self-healing concretes have been applied in real projects. Since 2015, more than ten self-healing concrete demonstration projects have been reported in the Netherlands, the United Kingdom, Belgium, China, and other countries. It was applied to a variety of environments, including tunnels, water channels, foundations, etc. [65,66,67].

H. M Jonker developed a self-healing concrete with the spores of bacillus and natural fibers, and he successfully applied it as lining for an irrigation canal in the highlands in Ecuador. There are no signs of cracking on the surface of the lining after one year [68,69]. A research project was carried out to construct mock retaining wall panels on an A465 valley highway project to investigate the development of self-healing cementitious materials. The perlite aggregates were fused with spores of bacillus psuedofirms species and organic nutrients (calcium acetate, yeast extract) under vacuum conditions and added to the concrete mixture as the self-healing agent. The panels were exposed to the environment in order to test the repair effect of the self-healing concrete in the actual project. After 6 months, the self-healing ability of panels was significantly improved [70].

Qian applied the produced microbial self-healing concrete to the side wall of the ship lock. It was observed that the cracking of the concrete was completely healed after 60 days, and the leakage of the crack was effectively blocked [71]. These projects indicated that the microbial self-healing concretes are expected to be widely used in concrete crack self-healing in hydraulic environments. Meanwhile, Qian et al. developed the batch preparation of spore powder by the spray-drying method and applied it into engineering construction. It effectively shortened the on-site construction time and promoted the development of microbial self-healing industrialization [71]. This technology has also been applied to the Nanjing-Jurong Intercity Rail Transit Project. The application position of the microbial self-healing concrete was located at the first layer of the underground structure. According to on-site observations, the self-healing concrete wall cracked 192 h after the completion of the construction. After covering the crack by a wet burlap with nutrient solution and water for 24 h, a large amount of white precipitates can be seen at the crack mouth. The crack covered by the wet burlap only generated a small amount of white precipitate; thus, in practical engineering applications, the timely addition of nutrients and moisture is an important factor in repairing cracks [65].

The current practical application of these microbial self-healing concretes provides a technical basis and reference for future large-scale usage (as Table 5 showing), but these application cases are still limited. The use of bacterial concrete in large-scale production is still affected by economic costs; more research is needed to reduce the production cost of the healing agents in order to boost the commercialization of microbial self-healing concrete.

## 6. Challenges for the Microbial Self-Healing Cement-Based Composites

### 6.1. Challenges of Microorganism in Concretes

There is limited ability for a certain number of microorganisms to decompose the substrate and generate calcium carbonate. Sufficient bacteria need to be mixed to repair cracks. However, if too many microorganisms are present, the death of the inactive microorganisms would have some effects on concrete, including the mechanical properties, the permeability of water, etc. If very few microorganisms are mixed, the need for fixing cracks cannot be fulfilled. Therefore, the problem of the relationship between the specific number of microorganisms in the organization and the number of cracks must be solved.

Nutrients are required components for microbial-mediated calcium carbonate precipitation in terms of microbial self-healing. However, the quantitative relationship between nutrients and microorganisms is still uncertain. The number of substrates would be abundant if microorganisms are insufficient, resulting in resource waste and an increase in cost. If the substrate is insufficient, the deposition of calcium carbonate will decrease, and the self-healing effect will deteriorate.

There is a limited lifetime of microorganisms in concrete. Aeration is one of the most important factors influencing the survival rate. If a technology can be developed or a mechanism can be discovered to increase the lifetime of concrete microorganisms, it is possible to increase the self-healing effect and reduce the repair costs of concrete fractures later on.

In concretes, there are mainly cracks on the exterior (not just on the surface) that have an effect on the whole. In this scenario, the procedure of surface brushing cannot fulfill the criteria for self-healing. However, in the concrete core, the carrier containing microorganisms and nutrients has only a limited repair impact, but the strength and permeability of concrete would have a great adverse effect. The self-healing impact of the central component of concrete, which can encourage advanced technologies and improve healing ability, can also be studied.

It is unknown about the real survival rate of encapsulated bacteria after being introduced to concretes and the lifestyles of embedded bacteria within the concrete matrix. Up to date, although there are a lot of reports to explain the internal self-healing process of microorganisms in cement-based composites, researchers just presented certain inferences. The internal self-healing process of repair still needs to be clarified. The activity range, the mode of activity, the consumption process of edible nutrients, the path of repair, the speed of repair, and the cause of changes in the speed of repair for activated concrete microorganisms are still unclear. If the repair mechanism of microorganisms can be understood at the micro stage, it can clarify the reason that the surface of the cracks may heal entirely under the uniform distribution of microorganisms in concrete, whereas the deep cracks heal just half. Then, the experiment operation would be improved in order to achieve high performance and complete crack repair.

The extent of a single microbial remediation is still unclear. If the scope of repair is precisely defined, the optimum self-healing effect can be accomplished by clarifying the optimal density of distribution of microorganisms on the basis that concrete performance is not affected by the carrier and nutrients. Or even if the carrier and nutrients have an effect on the concrete’s efficiency, the optimal self-healing effect can be calculated by a comprehensive consideration of the optimal density of microbial distribution. It is important to explain the relationship between the certain amounts of calcium carbonate produced by microorganisms and the producing time, and it is crucial to analyze the distribution type of calcium carbonate produced by microorganisms at the micro level. Whether calcium carbonate accumulates only in one direction or in all directions, which can determine the microbe quantity and achieve the best self-healing effect must be determined.

The electro-hydraulic servo testing machine, compressive servo testing machine, automated bending compression testing machine, fishing line, iron nail, and other techniques are used by many academics in experimental research to generate cracks. However, in practical engineering, these man-made cracks are not the same as the cracks occur naturally. Therefore, it is important to make the same crack as the actual situation, which can increase the effective rate of crack repair in practical applications in engineering.

The laboratory environment is the primary environment in which self-healing processes are studied. However, the concrete should be used in very different environments, such as marine water, rivers, soil, or frozen soil. In addition, it is necessary to consider some special situation, for example, the freeze–thaw cycles. Water can enter the concrete and dissolve the concrete matrix in each freezing period and thawing cycle due to changes in internal and external pressures. In this situation, the assessment of the effectiveness of a healing agent’s consolidation capacity is essential. Therefore, in order for practical engineering to have a great self-healing effect, these conditions should be simulated during research. The performance of microorganisms in various conditions should receive more consideration, and the final repair effect should be tested in the laboratory process.

In addition to the performance of the self-healing process, the current production cost of the bacterial self-healing agent is also a challenge. To satisfy economic constraints, improved production methodologies will need to be developed, particularly with regard to the large-scale cultivation of bacteria, nutrients, and labor intensity. Strategies to increase bacterial self-healing efficiency and reduce costs need to be developed, which will accelerate the adoption of MICP-applied concrete in the future.

### 6.2. Fundamentals for Further Investigation

In the field of microbial self-healing, due to the strong alkaline and poor living environment in concrete interiors, it is necessary to identify a microorganism with tenacious vitality, can resist strong alkalinity, and has an excellent repair effect. Otherwise, the interior of concrete is basically an anaerobic environment, so anaerobic bacteria will be more suitable for concrete self-healing. In addition, the mixed bacteria have more tenacious survival ability and can complete the task better, so we can use two or more microorganisms mixed in a certain proportion to make a mixed bacterium and try to study the repair effect. Genetic modification is also feasible, inserting exogenous DNA to achieve the necessary properties of the microorganisms needed for self-healing concrete. At the same time, the main reason for the lack of large-scale application of microbial self-healing concrete is its high production cost, especially the high cost of the cultivation of microorganisms and the use of nutrients. Therefore, it is necessary to find a lower cost of microbial culture methods and relatively cheap nutrients where the technology can have a better and wider application.

The fundamental purpose of the carrier is to protect the survival of microorganisms under mechanical stress and high alkaline environment. In order to provide an excellent living environment and place for microorganisms, the carrier should have high porosity and great biocompatibility. The acquisition cost and application cost of the carrier should also be evaluated. In addition, the choice of carrier needs to consider the practical use of concrete. The performance of the level of concrete in different facilities and structures are different. Each structure has special features in some performance aspects and the effect carriers showed ultimately is different. Therefore, it is necessary to develop specific carriers for microbial self-healing materials with a series of special structures to exploit the structural strengths of each carrier. In the future, it will be important to improve the microbial immobilization methods or develop a new one that is easy to operate with higher efficiency.

## 7. Conclusions

This paper focused on three main factors in the self-healing cement concretes including microorganisms, carriers, and microbial immobilization methods. The main conclusions are drawn as follows.

(1) The species and survival rate of microorganisms in microbial concrete materials need to be optimized. The microorganisms can be divided into urease bacteria and non-urease bacteria. Urease bacteria precipitate CaCO_3_ crystals through a series of reactions, whereas non-urease bacteria convert organic acids into CaCO_3_ precipitates through their own hypoxic reactions. However, ammonia gas will be produced in this process when using urease bacteria, which is very undesire in artificial building materials. In the future direction of development, it is necessary to seek a more green and scientific way to fix ammonia, or through gene modification, insert exogenous DNA to achieve the necessary properties of microorganisms needed for self-healing concrete, such as alkalinity resistance, anaerobic, environmental protection, etc. The cultivation and nutrients of microorganisms account for a large part of the cost of microbial concrete materials. To realize the economical and practical use of this material, it is necessary to find a cheaper cultivation method and nutrients.
(2) The microbial carrier is an important factor that affects the self-healing efficiency of microbial concrete materials, which deserves more research. At present, carriers are mainly divided into organic carriers and inorganic carriers, which can work in different applications. Choosing a suitable carrier in the corresponding environment can improve the efficiency of self-healing to a certain extent. Despite the fact that the carrier can prolong the survival time of microorganisms in an extremely alkaline environment, the successful operation time of existing bacteria is still insufficient compared with actual engineering applications. It is necessary to develop more bio-affinity, high stability, and good compatibility carrier materials—for example, by changing the surface group or micropore composition of the carrier material, which can promote the production and application of self-healing concrete technology.
(3) Microbial immobilization is an indispensable step in making microorganism self-healing concrete. Although there are many microbial immobilization methods at present, all of the methods and preparation conditions have corresponding standards, and each method has its own characteristics and merits. This paper focuses on the analysis of three commonly used methods which include ATM, MEM, and VIM and lists their repair effect. When the same microorganisms, carriers, and nutrients are used, and other variables are controlled the same, it will not have much impact on the final repair effect by changing the microbial immobilization method only. During microbial immobilization, special attention should be paid to the experimental operation process and method to avoid the decrease of the success rate of immobilization caused by unscientific and unprecise experiments, which will impact the final repair effect.
(4) The microbial self-healing materials in the specific repair effect of microorganisms, survival ability, impact factor, and lifestyle in the concrete matrix are still unclear. The real application of self-healing concrete in civil engineering is still limited, and standardized methods for evaluate the healing capacity have not been established. The survival time of microorganisms in the concrete is limited. The current technology makes it difficult to know the actual survival rate of the bacteria after being introduced into the concrete. It is also difficult to determine the optimal distribution density of microorganisms. The research on the internal repair mechanism of concrete contributes to the development of more economical and practical microbial concrete.

## Figures and Tables

**Table 1:** Calcium carbonate precipitation reaction in bacterial urease.

Bio-Chemical Reactions	Equation
CO(NH_2_)_2_ + H_2_O → NH_2_COOH + NH_3_	(1)
NH_2_COOH + H_2_O → NH_3_ + H_2_CO_3_	(2)
2NH_3_ + 2H_2_O ↔ 2NH_4_^+^ + 2OH^−^	(3)
H_2_CO_3_ ↔ HCO_3_^−^ + H^+^	(4)
HCO_3_^−^ + H^+^ + 2OH^−^ ↔ CO_3_^2−^ + 2H_2_O	(5)
Ca^2+^ + *Cell* → *Cell* − Ca^2+^	(6)
*Cell* − Ca^2+^ + CO_3_^2−^ → *Cell* − CaCO_3_ ↓	(7)

**Table 2:** Optimum pH value and maximum repaired crack width of different microorganisms.

Bacteria	PH	Repairing Effects	Ref.
Bacillus cohnii	11.0	Maximum repaired crack width is 0.56 mm, compressive strength increases by 30%.	[21]
Bacillus pasteurii	9.25	Maximum repaired crack width is 3.8 mm, compressive strength increases by 40%.	[22,26,30]
Bacillus pseudofirmus	11.0	CaCO_3_ precipitation content is 86%.	[21]
Bacillus sphaericus	7.0	Maximum repaired crack width is 0.97 mm, CaCO_3_ content grows up with time.	[23,27,28,31]
Alkaliphilic Bacillus	——	Maximum repaired crack width is 0.46 mm, CaCO_3_ precipitation content is 94.32%.	[30,32,33]
Bacillus pseudofirmus	8.0	Maximum repaired crack width is 1.8 mm, 22% increase of compressive strength, 7% increase of flexural strength.	[34,35]

**Table 3:** Influence by carriers on the strength and repairing width of self-healing concrete.

Carrier Materials			Strength	Maximum Value of Completely Healed Crack Width/mm	Ref.
Inorganic material	Natural	Diatomite	/	0.17	[13]
		Pelelith	Increase	2	[43]
	Artificial	Expanded perlite	/	0.79	[39]
		Expanded clay	/	0.45	[39]
		Porous ceramist	Decrease	0.51	[41]
		Zeolite	Increase	/	[42]
Organic material	Natural	Agar	Decrease	0.1	[46]
		Odium alginate	Decrease	0.1	[46]
	Artificial	Microcapsule	Decrease	0.97	[48]
		Graphite nano platelets	Increase	0.52	[50]

**Table 4:** The maximum repaired width of microorganisms with different immobilization methods and carriers.

Immobilization	Carrier	Microorganism	Maximum Repaired Width/mm	Ref.
Adsorption treatment method (ATM)	Expanded perlite	Bacillus aerobic alcalophilus	0.79	[56]
	Ceramsite	new microbe	0.3	[55]
	Diatomaceous earth	LMG22557	0.17	[13]
	Pelelith	Bacillus pasteurii	2	[43]
	Expanded perlite	Bacillus cohnii	0.56	[20]
Microcapsule embedding method (MEM)	Microcapsule	LMG22557	0.97	[48]
	Microcapsule	Bacillus cohnii	0.1	[57]
Brushing method	Agar	Carbonic anhydrase microorganism	0.1	[60]
Vacuum impregnation method (VIM)	Expanded clay particles	bacterial spore	0.46	[58]
	Expanded clay particles	Bacillus cohnii	0.45	[39]
	Expanded perlite	Bacillus cohnii	0.79	[39]
	Expanded perlite	Bacillus cohnii	1.22	[59]
Vacuum saturation method (VSM)	Granular activated carbon	Diaphorobacter nitroreducens/Pseudomonas aeruginosa	0.48	[61]
	Porous ceramsite particles	Bacillus pasteurii	0.45	[62]

**Table 5:** Summary of practical application of microbial self-healing concrete in civil engineering.

Self-Healing Agents	Application Environments	Application Effect	Ref.
Powder-based healing agent	Underground structure in Nanjing-Jurong Intercity Rail Transit Project	Part of white precipitates were formed on the surface of the crack after 28 days	[65]
MUC^+^ (Mixed Ureolytic Culture and anaerobic granular bacteria)	A roof slab of an inspection chamber of one of the drainage pipes	An inspection of the roof slab showed no signs of cracking, yet favorable conditions for healing were observed	[66]
A mixture spores of alkali-resistant bacteria and a food source for the bacteria: Calcium lactate and yeast extract	An irrigation canal	No signs of cracking on the surface of the lining after one year	[68,69]
Spores of bacillus psuedofirms species, and organic nutrient (calcium acetate, yeast extract)	A mock retaining wall panels on A465 valley Highway project	Significant improvement in self-healing of panels after 6 months	[70]
Bacteria spore powder and calcium source	The Mangdao River ship lock	The connectivity of the cracks had been completely blocked, and no more water leaked after 65 days	[71]

## Data Availability

Data available upon request from the corresponding author.
